# Ketones Prevent Oxidative Impairment of Hippocampal Synaptic Integrity through K_ATP_ Channels

**DOI:** 10.1371/journal.pone.0119316

**Published:** 2015-04-07

**Authors:** Do Young Kim, Mohammed G. Abdelwahab, Soo Han Lee, Derek O’Neill, Roger J. Thompson, Henry J. Duff, Patrick G. Sullivan, Jong M. Rho

**Affiliations:** 1 Departments of Neurology and Neurobiology, Barrow Neurological Institute, St. Joseph’s Hospital & Medical Center, Phoenix, Arizona, United States of America; 2 Departments of Cell Biology and Anatomy, Hotchkiss Brain Institute, University of Calgary, Calgary, Alberta, Canada; 3 Libin Cardiovascular Institute of Alberta, University of Calgary, Calgary, Alberta, Canada; 4 Spinal Cord and Brain Injury Research Center, University of Kentucky, Lexington, Kentucky, United States of America; 5 Departments of Pediatrics and Clinical Neurosciences, Alberta Children’s Hospital, Calgary, Alberta, Canada; Xuzhou Medical College, CHINA

## Abstract

Dietary and metabolic therapies are increasingly being considered for a variety of neurological disorders, based in part on growing evidence for the neuroprotective properties of the ketogenic diet (KD) and ketones. Earlier, we demonstrated that ketones afford hippocampal synaptic protection against exogenous oxidative stress, but the mechanisms underlying these actions remain unclear. Recent studies have shown that ketones may modulate neuronal firing through interactions with ATP-sensitive potassium (K_ATP_) channels. Here, we used a combination of electrophysiological, pharmacological, and biochemical assays to determine whether hippocampal synaptic protection by ketones is a consequence of K_ATP_ channel activation. Ketones dose-dependently reversed oxidative impairment of hippocampal synaptic integrity, neuronal viability, and bioenergetic capacity, and this action was mirrored by the K_ATP_ channel activator diazoxide. Inhibition of K_ATP_ channels reversed ketone-evoked hippocampal protection, and genetic ablation of the inwardly rectifying K^+^ channel subunit Kir6.2, a critical component of K_ATP_ channels, partially negated the synaptic protection afforded by ketones. This partial protection was completely reversed by co-application of the K_ATP_ blocker, 5-hydoxydecanoate (5HD). We conclude that, under conditions of oxidative injury, ketones induce synaptic protection in part through activation of K_ATP_ channels.

## Introduction

The ketogenic diet (KD), a proven treatment for medically intractable epilepsy [1], results in prominent production of ketones (notably, D-β-hydroxybutyrate [BHB] and acetoacetate [ACA]). Increasingly, ketones have been shown to exert neuroprotective actions in models of neurodegenerative disorders (NDs), likely by restoration of impaired mitochondrial metabolism and antioxidant capacity [2,3]. A common pathogenic feature of NDs is oxidative stress which correlates closely with progressive tissue injury, and when the hippocampus is affected, results in cognitive and memory deficits. Conversely, a synthetic BHB ester-linked polymer, KTX-0101, and dietary ketosis are shown to mitigate memory deficits in patients of Alzheimer’s disease (AD) [4,5] In line with this, ketones attenuate impairment of hippocampal cognitive function in a model of AD [6]. Further, *in vitro* studies have demonstrated a crucial role for ketones in preserving hippocampal synaptic integrity in the face of oxidative stress and mitochondrial dysfunction [3,7]. However, the specific mechanisms underlying their beneficial actions remain unclear.

ATP-sensitive potassium (K_ATP_) channels act as metabolic sensors, coupling cellular metabolism with neuronal activity by enhancing K^+^ efflux. It is well known that plasmalemmal or surface K_ATP_ (sK_ATP_) channels are activated under conditions of metabolic stress [8–10]. K_ATP_ channels localized to the mitochondrial inner membrane (i.e., mitoK_ATP_ channels) may regulate mitochondrial homeostasis by modulating electron transport and calcium buffering [11]. Both sK_ATP_ and mitoK_ATP_ channels have been implicated in models of tissue injury, notably in the heart and brain [11–14]. Activation of these channels may inhibit mitochondrial permeability transition (mPT), a critical determinant of cell death [15–18]. Recently, Yellen and colleagues reported that ketones inhibit spontaneous firing of substantia nigra pars reticulata neurons through sK_ATP_ channels, likely by increasing their open probability [19,20]. In the present study, we sought to determine whether the functional neuroprotective effects of ketones against hydrogen peroxide (H_2_O_2_)-induced impairment of hippocampal long-term potentiation (LTP) are mediated through either sK_ATP_ and/or mitoK_ATP_ channels.

## Materials and Methods

### Preparation of hippocampal slices and electrophysiology

All animal handling protocols were approved by the Institutional Animal Care and Use Committees at the Barrow Neurological Institute (Protocol number 405/308) and the University of Calgary (AC11-0047). Transverse hippocampal slices (400-μm thickness) were prepared from brains of 5- to 6-week-old Sprague Dawley rats or Kir6.2KO mice (Kir6.2^−/−^). Age-matched Kir6.2^+/+^ mice (wild-type mice; WT) were used as controls. Following decapitation, the whole brain was rapidly isolated and submerged in ice-cold oxygenated physiological saline (composition in mM: 124 NaCl, 1.8 MgSO_4_, 4 KCl, 1.25 NaH_2_PO_4_, 26 NaHCO_3_, 2.4 CaCl_2_, and 10 D-glucose; pH: 7.4). Slices were cut using a vibratome (The Vibratome Company, St. Louis, MO), and then transferred to an incubation chamber containing physiological saline bubbled with 95% O_2_/5% CO_2_ at 35°C for 1 hr. Each slice was transferred to a submersion-type recording chamber affixed to a Zeiss AxioSkop FS2 microscope and superfused with warm (31 ± 1°C) physiological saline at a rate of 2–3 ml/min before the start of each experiment. To measure changes in synaptic transmission, extracellular population spikes (PS) were evoked by stimulation of Schaffer collaterals (SC) using a bipolar concentric electrode, and responses were recorded in stratum pyramidale of CA1 with a recording electrode (2–6 MΩ tip resistance, backfilled with 2 mM NaCl) connected to a Multiclamp 700A amplifier and digitized with a Digidata 1322A (Axon Instruments). The electrical stimulus was a 100 μs pulse with an intensity (20–100 μA) set at 50% of maximal PS amplitude. After obtaining a stable evoked PS, changes in PS amplitude following application of reagents were digitally stored for later off-line analysis. Upon SC stimulation, excitatory postsynaptic potentials (EPSP) were recorded at a control test frequency of 0.05 Hz (0.1ms, 20–100 μA) from stratum radiatum of CA1 hippocampus. An input-output curve (i.e., stimulus intensity *vs*. EPSP amplitude) was constructed and the baseline EPSP amplitude (over 1 mV) was set to 30–40% of the maximum response. To measure changes in synaptic plasticity, long-term potentiation (LTP) was evoked by theta-burst stimulation (TBS) consisting of 5 spike trains delivered at 0.2 Hz (each train, five stimuli at 0.1ms, 100 Hz). LTP is a form of synaptic plasticity broadly used as an electrophysiological measure of learning and memory. LTP was expressed as the percent of mean baseline EPSP amplitude. Paired-pulse facilitation (PPF) was tested to measure changes in presynaptic functions [21]. The interval between two stimuli varied from 50 to 100, 200, and 300 ms. Following PPF, EPSP amplitude was normalized by indicating the ratio (second EPSP/first EPSP). Recorded data were filtered at 3 kHz, sampled at 10 kHz using pClamp, and analyzed with Clampfit.

### HT-22 cells and cell viability test

Murine hippocampal HT-22 cells were provided as a gift by Dr. Richard Dargusch (The Salk Institute)[22] and fed with Dulbecco's Modified Eagle Medium (DMEM) supplemented with 10% fetal bovine serum (FBS), 100 U/mL penicillin, and 100 mg/mL streptomycin. Cells were maintained in a humidified incubator with 5% CO_2_ at 37°C. HT-22 cells were seeded at a density of 4,000 cells per well in 96-well cell culture plates and grown overnight. Subsequently, cells were pretreated with either ketone alone (BHB or ACA; each 1 to 3 mM) or a ketone cocktail (BHB and ACA; each 1 mM) overnight followed by the addition of H_2_O_2_ (200 μM to 1 mM) for 3, 6, 9, 12, and 24h. Cell viability after H_2_O_2_ administration was evaluated by the 3-(4,5-dimethylthiazol-2-yl)-2,5-diphenyltetrazolium bromide (MTT) reduction assay. Twenty μl of MTT (2.5 mg/ml) was added to the wells and incubated for 1h. After incubation, the medium was removed from the wells and 100 μl of dimethylsulfoxide (DMSO) was added to dissolve the formazan reagent. The absorbance was read using a microplate reader at 565 nm. Cell viability was expressed as a percentage of the control group.

### ATP assay

Tissue samples were obtained as previously described from the CA1 region of rat brain slices (n = 12 in each experimental group from five rats) using 18-gauge needles [3]. Briefly, a mixture of each sample in 5% trichloroacetic acid (100-fold dilution, volume/weight) was sonicated for 10 min and then centrifuged at 14,000 g for 2 min at 4°C. Ten μl of supernatant from each sample was diluted 1,000-fold in phosphate-buffered saline and then mixed with the Enlighten ATP assay kit reagents (Promega, Medison, WI, USA) according to the manufacturer’s instructions. Light intensity after final reaction was measured in a TD-20/20 Luminometer (Turner Biosystems, Sunnyvale, CA, USA) and then compared to an ATP standard curve collected from serial dilutions of an ATP stock solution.

### Drugs and statistical analysis

All reagents used in this study were purchased from Sigma-Aldrich (St. Louis, MO), unless stated otherwise. Hydrogen peroxide (H_2_O_2_) was made fresh for each trial from stock solution; D-β-hydroxybutyrate (BHB); acetoacetate (ACA); and 5-hydroxydecanoate (5-HD) were also prepared immediately before each experiment and directly dissolved in physiological saline. While ACA is known to be spontaneously decarboxylated to acetone, once dissolved in saline, it is not volatile. Diazoxide (DZ) was added to physiological saline as a 20 mM stock solution dissolved in NaOH (0.1 M), after which the pH was adjusted to 7.4 with HCl. Glibenclamide (Glb) was dissolved in dimethyl sulfoxide (DMSO). Numerical data are expressed as the mean ± SEM for n slices. Student t-test or ANOVA were performed to determine the presence of significant variance in data from different experimental groups. *p* <0.05 was considered to be significant.

## Results

### Ketones preserve synaptic integrity against hydrogen peroxide (H_2_O_2_)

**Fig 1:**
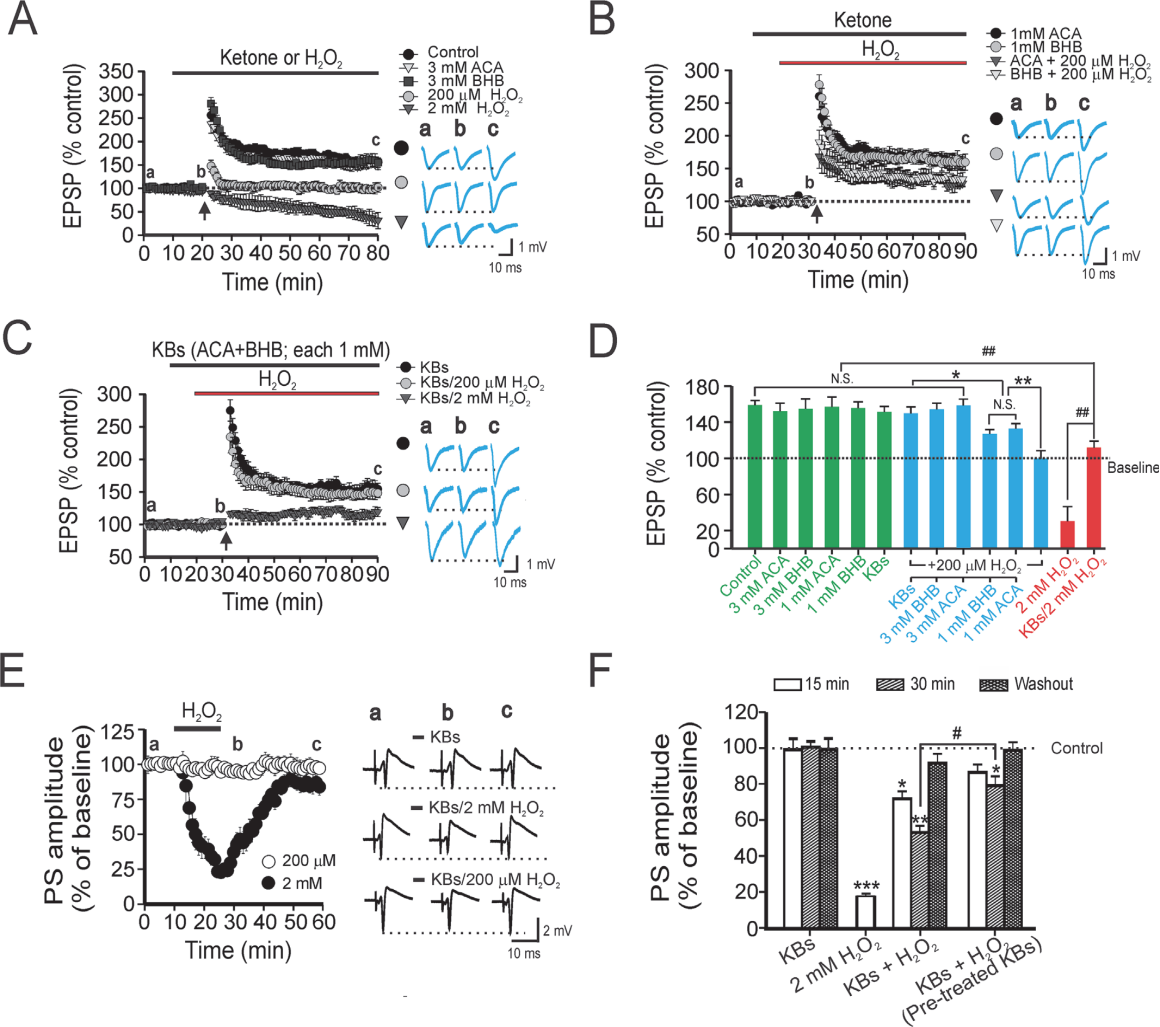
Ketones ameliorate synaptic impairment caused by hydrogen peroxide (H_2_O_2_). (A) LTP recorded in rat CA1 hippocampal slices exposed to various concentrations of H_2_O_2_. Theta-burst stimulation (TBS) of schaffer collaterals led to robust LTP changes when either ACA or BHB (3 mM each) were perfused, but not with either 200 μM or 2 mM H_2_O_2_. Representative traces of excitatory post-synaptic potentials (EPSPs) at respective time-points (a, b, c) depicted on the right of each panel. Vertical arrows in this and following figures indicate the time-point of TBS initiation. Dotted line denotes the baseline field potential amplitude which is the mean EPSP during a 10 min physiological saline infusion. (B) Only partial inhibition of LTP was observed when 200 μM H_2_O_2_ was co-applied with either ACA or BHB (1 mM each). (C) Intact LTP formation was seen when a cocktail of ketones (BHB and ACA, 1mM each) was used in conjunction with 200 μM H_2_O_2_, but not with 2 mM H_2_O_2_. (D) Summary bar graph indicating changes in EPSP amplitudes at 60 min after TBS amongst various treatment groups. Dose-dependent protection by ketones against LTP impairment by H_2_O_2_-induced oxidative stress was seen in these experiments. Each vertical bar represents the EPSP amplitude ± SEM (obtained in 10 slices from 5 rats). One way ANOVA followed by Tukey *post-hoc* analysis; *, *p* <0.05; ** or ^##^, *p* < 0.01, NS, not significant. (E) Acute application of H_2_O_2_ results in depression of the population spike (PS) in stratum pyramidale of Cornu Ammonis (CA)1. Shown are changes in the mean (± SEM) PS amplitude before, during, or after H_2_O_2_ infusion, but only when 2 mM H_2_O_2_ was used. Representative traces of the PS alone (*top*), or co-applied with 2 mM H_2_O_2_ (*middle*) or 2 mM H_2_O_2_ (*bottom*) depicted on the right. Pre-incubation with ketones potentiated the restoration of PS amplitude compared to co-application of ketones with H_2_O_2._ (F) Summary of amplitude changes in the PS (reflected as % of baseline) during or after drug application. Each vertical bar represents PS amplitude ± SEM, and data were collected from 12 slices from 5 rats. The dotted line reflects the baseline (100%) control PS amplitude before drug treatment. Asterisks denote significant differences between control and treatment groups (*, *p* < 0.05; ** *p* < 0.01; ***, *p* < 0.001), whereas # indicates significant differences between the ketones plus H_2_O_2_ group, and other groups in which pretreatment of ketones occurred before H_2_O_2_ application (*p* < 0.05).

Hippocampal slices from normal rats displayed robust LTP following theta-burst stimulation (TBS) in physiological saline. The EPSP amplitude measured 256 ± 13% and 159 ± 5% at peak and 60 min post-TBS, respectively (**Fig. 1A**). Perfusion of 2 mM H_2_O_2_ in hippocampal slices gradually led to a decrease in baseline EPSP amplitude, and an eventual failure of TBS to induce LTP (**Fig. 1A, D**). Interestingly, while the baseline EPSP amplitude was not altered in response to 200 μM H_2_O_2_, LTP was still impaired (**Fig. 1A**). Thus, in hippocampal slices exposed to H_2_O_2_, the EPSP amplitude at 60 min post-TBS was significantly decreased compared to the control group (*p*<0.01, **Fig. 1D**), confirming that exogenous application of H_2_O_2_ disrupts hippocampal synaptic plasticity in a dose-dependent manner.

Having established the adverse effects of H_2_O_2_, we then examined the effects of ketones on oxidative impairment of hippocampal LTP. Since brain and serum ketone levels have been reported to be in the range of 0.5–5 mM during the suckling period in immature rodents and during treatment with the KD in humans [23–26], we used a similar concentration range (BHB or ACA; each 1 to 3 mM) in this study. In hippocampal slices exposed to ketones alone, no differences were seen in the EPSP amplitude between control and ketone-applied groups at 60 min post-TBS (**Fig. 1A, B, D**). When a ketone alone (BHB or ACA; 1 mM each) was co-applied with 200 μM H_2_O_2_, LTP was only partially inhibited; the EPSP amplitude of these two groups at 60 min post-TBS was significantly decreased compared to that of controls (*p*< 0.05, **Fig. 1B, D**). In contrast, LTP was sustained following co-application of a ketone cocktail (ACA and BHB, each 1 mM) and 200 μM H_2_O_2_; there was no difference between the EPSP amplitude of ketone-treated hippocampal slices compared to controls (**Fig. 1C, D**). And as expected, the deficit in LTP induced by 200 μM H_2_O_2_ was reversed by 3 mM ketone (**Fig. 1D**). Despite the slightly increased EPSP amplitude post-TBS compared to 2 mM H_2_O_2_ alone, LTP impairment was maintained in the presence of a cocktail of ketones and 2 mM H_2_O_2_ (**Fig. 1C, D**).

Next, we determined whether ketones would preserve normal hippocampal synaptic transmission when challenged with H_2_O_2_. Synaptic transmission was progressively depressed during perfusion of 2 mM H_2_O_2_; the PS amplitude measured 18 ± 3% of baseline after a 15 min infusion with 2 mM H_2_O_2_, and then recovered to 84 ± 5% of baseline following a 30 min washout (**Fig. 1E**). Despite a clear deficit in LTP produced by 200 μM H_2_O_2_, changes in PS amplitude were not seen in the presence of 200 μM H_2_O_2_ (**Fig. 1E**). Further, no change in the PS amplitude was found with an infusion of a ketone cocktail (**Fig. 1E, F**). Upon application of a ketone cocktail with 2 mM H_2_O_2_, ketones prevented the synaptic depression under conditions of H_2_O_2_ exposure; the evoked PS amplitude measured 72 ± 4% and 59 ± 5% of baseline at 15 min and at 30 min time-points, respectively. Additionally, a 30-min washout restored the PS amplitude to 92 ± 5.4% of baseline (**Fig. 1E, F**). The synaptic protection was potentiated in hippocampal slices with a 10 min pre-incubation with ketones (**Fig. 1F**). The PS amplitude of the ketone pre-incubated group was significantly different than that seen when a ketone cocktail and H_2_O_2_ were co-applied after a 30 min infusion (*p*< 0.05; **Fig. 1F**). These data indicate that physiological concentrations of ketones can mitigate hippocampal synaptic depression under conditions of oxidative stress.

### Activation of K_ATP_ channels provides synaptic protection against H_2_O_2_

**Fig 2:**
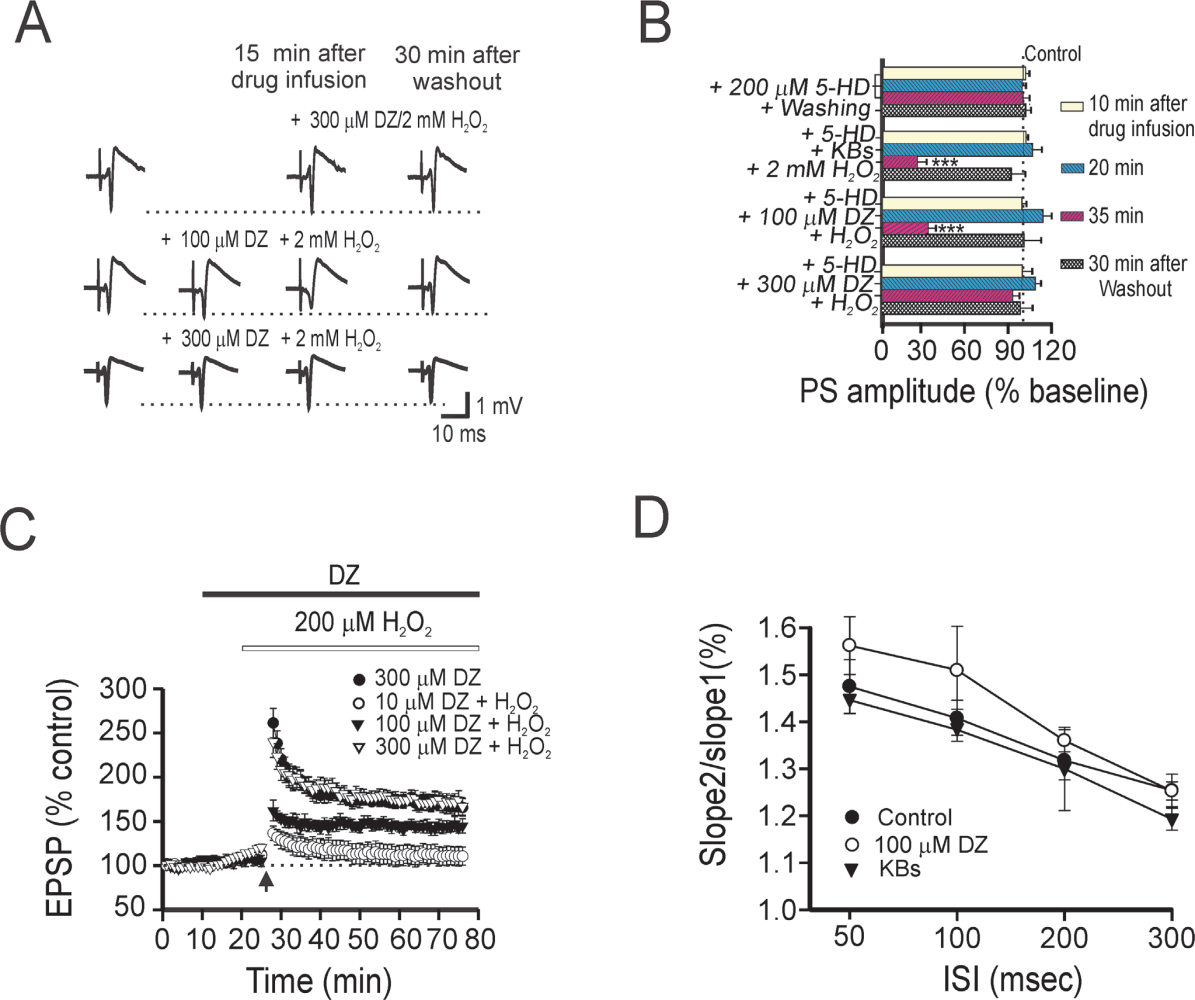
Diazoxide mimics the synaptic effect of ketones. (A) Representative traces of DZ (100 μM or 300 μM) with 2 mM H_2_O_2_ on the CA1 hippocampal PS, demonstrating the dose-dependent synaptic protective effects of DZ. Partial protection was seen with H_2_O_2_ exposure following a 10 min pre-incubation with 100 μM DZ (*middle*). No significant differences were found between the two groups (co-application *vs*. pre-incubation) compared to 300 μM DZ. (B) Summary of PS amplitude changes (as % baseline) during or after drug application is shown in the bar graph. Each vertical bar indicates mean PS amplitude ± SEM, and was obtained in 12 slices from 5 rats. Asterisks denote significant differences between the 100 μM DZ plus 2 mM H_2_O_2_ group and other treatment groups (**, *p* < 0.01). (C) DZ protects synaptic impairment against oxidative stress. Representative traces of EPSPs at respective time-points (a, b, c) are depicted on the right. (D) Changes in paired-pulse facilitation at CA1 in hippocampal slices after 40 min infusion of physiological saline, KBs (a cocktail of BHB and ACA; each 1 mM) and 100 μM DZ. Exposure to either KBs or DZ did not induce a significant change in presynaptic transmission following paired-pulse stimulation. The data was obtained in 7 slices from 4 mice. ISI indicated interstimulus interval.

Diazoxide (DZ) is an activator of both sK_ATP_ and mitoK_ATP_ channels, and has been demonstrated to exert cellular protective effects in pre-ischemic preconditioning and against oxidative injury [16,27]. While both types of K_ATP_ channels have clearly been shown to mediate neuroprotective actions under various pathological conditions, their effects on synaptic neurotransmission have yet to be fully elucidated. To investigate whether K_ATP_ channels affect synaptic transmission, we tested the effects of DZ on the reduction in PS amplitude caused by H_2_O_2_. Upon co-application of 300 μM DZ plus 2 mM H_2_O_2_, the PS amplitude remained constant and similar to control values (**Fig. 2A, B**). Further, when DZ was pre-incubated with 300 μM H_2_O_2_ for 10 min, no differences in PS amplitude were seen compared to controls (**Fig. 2A**). Pre-incubation with 100 μM DZ also prevented PS depression caused by H_2_O_2_, but the recovery rate of the PS amplitude was significantly slower than that seen with 300 μM DZ (*p*< 0.05). The PS amplitude measured 62 ± 3% of control at 15 min post 100 μM DZ plus H_2_O_2_ co-application, and then recovered to 92 ± 6% of control after a 30 min washout (**Fig. 2A, B**). Taken together, these results show that DZ can exert protective effects against H_2_O_2_-induced impairment of the hippocampal PS.

To determine whether K_ATP_ channels can influence hippocampal synaptic plasticity during oxidative stress, we measured the effects of DZ (10 μM to 300 μM) on LTP impairment induced by 200 μM H_2_O_2_. Normal LTP was sustained at hippocampal synapses when exposed to 300 μΜ DZ alone or when 300 μΜ DZ and H_2_O_2_ were co-applied the EPSP amplitude at 50 min post-TBS measured 178 ± 15% and 172 ± 10%, respectively (**Fig. 2C**). However, lower concentrations of DZ were unable to fully prevent H_2_O_2_-induced LTP impairment; the EPSP amplitude of the 100 μM DZ plus H_2_O_2_ group measured 162 ± 12% and 142 ± 7% at peak and at 50 min post-TBS, respectively (**Fig. 2C**). And 10 μM DZ had no effect on the reduced EPSP amplitudes caused by 200 μM H_2_O_2_. Paired-pulse facilitation (PPF) did not alter at hippocampal synapses when a KB cocktail (BHB and ACA; 1 mM each) was applied, compared to physiological saline alone (**Fig. 2D**). When 100 μM DZ was infused, the EPSP amplitude of the second response relative to the first resulted in a slight increase in facilitation. However, one-way ANOVA did not find any overall difference amongst the experimental groups. Collectively, these data indicate that the activation of K_ATP_ channels in response to DZ can suppress synaptic impairment in oxidative stress, leading to restoration of synaptic integrity.

### Functional neuroprotection induced by ketones arises from K_ATP_ channels opening and ATP generation

**Fig 3:**
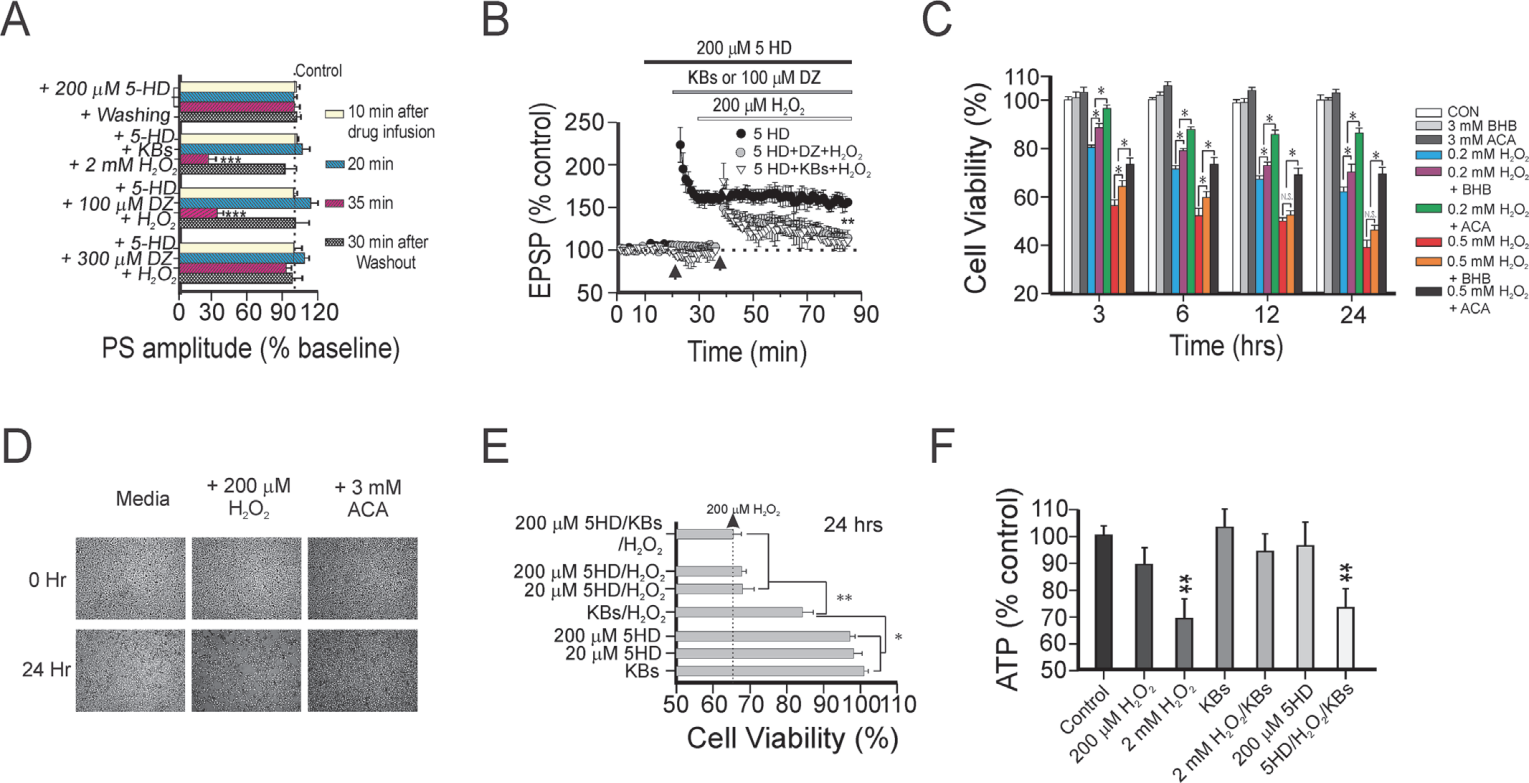
Pharmacological blockade of K_ATP_ channels negates the hippocampal synaptic and neuronal protection afforded by ketones. (A) Changes in PS amplitude (as % of baseline) during or after drug application under different experimental conditions. No changes were seen in the PS amplitude during infusion with 200 μM 5HD alone or 5HD with 300 μM DZ and H_2_O_2_. There was a reversible, dose-dependent blockade by 5HD of the PS when either ketones or 100 μM DZ were applied concurrently with H_2_O_2_. Asterisks denote significant differences between experimental groups and the control at a specific time-point (*** *p* < 0.001). The dotted line indicates the mean PS amplitude of the control group. Each horizontal bar indicates mean PS amplitude ± SEM obtained in 12 slices from 5 rats. (B) Normal TBS-induced LTP was seen with 200 μM 5HD. However, LTP was impaired when either ketones or 100 μM DZ were administered together with 5-HD; the EPSP amplitude was measured 110 ± 9% and 115 ± 11% at 50 min post-TBS, respectively. Asterisks denote significant differences between 200 μM 5-HD alone and other treatment groups (**, *p*<0.01). Each LTP dataset was collected in 10 hippocampal slices. (C) Time-course of the MTT assay for cell viability in murine hippocampal HT22 cells treated with H_2_O_2_—with or without application of BHB or ACA (each 3 mM)—for 3, 6, 12, and 24 hours. Each vertical bar indicates the mean ± SEM (n = 30). (D) Representative photomicrographs of HT22 cells under control conditions, with H_2_O_2_, or when H_2_O_2_ was co-treated with ACA at two time-points (0 and 24 hrs). (E) Oxidative-induced cell death in HT22 cells measured under different treatment conditions. While 20 μM and 200 μM 5HD alone had no influence on cell viability, ketone (each 1 mM)-mediated neuronal protection against oxidative injury was completely negated by 200 μM 5HD. (F) Bar graphs illustrating changes in ATP levels in hippocampal CA1 samples treated with ketones with or without 5-HD on H_2_O_2_-induced oxidative stress after 2 hrs. ATP levels represented as % of control are mean ± SEM of 12 slices analyzed after 2 hr with indicated significant decreases (**p < 0.01) compared with control group, which was infused with physiological saline under similar experimental conditions.

A recent study has shown that ketones can control neuronal firing *via* K_ATP_ channels [28]. These observations, together with a previous finding that pharmacological blockade of K_ATP_ channels with 5-hydoxydecanoate (5HD) abolished DZ-mediated synaptic protection [29], led us to investigate whether the synaptic protection provided by ketones against oxidative stress could be reversed by 5HD. When hippocampal slices were exposed to 200 μM 5HD alone, no changes in the PS amplitude were seen (**Fig. 3A**). However, a serial infusion of 200 μM 5HD that included a cocktail of ketones (ACA and BHB, 1 mM each) and 2 mM H_2_O_2_ at 10 min intervals resulted in a tendency toward synaptic depression. 5HD exerted a similar blockade when 100 μM DZ was used, but increasing the DZ concentration to 300 μM negated this action of 5HD (**Fig. 3A**). After application of 200 μM 5HD, there was no detectable change in LTP formation (**Fig. 3B**). However, pre-treatment with 5HD abolished the ketone-induced preservation of LTP: LTP measured 181 ± 23% and 110 ± 9% at peak and at 60 min post-TBS, respectively. Further, LTP was not sustained when 5HD was added to 100 μM DZ and H_2_O_2_; LTP measured 115 ± 11% at 60 min post-TBS (**Fig. 3B**). These results differed significantly from both control conditions and with 200 μM H_2_O_2_ alone (*p* < 0.01).

Following these observations, we measured changes in the viability of murine hippocampal HT22 cells to investigate long-term effects of ketones against H_2_O_2_–induced oxidative stress. While H_2_O_2_ application for 3, 6, 12, and 24 hrs dose-dependently increased cell death, these effects were significantly ameliorated by pre-treatment with ketones (BHB or ACA; each 1 mM or 3 mM) (**Fig. 3C**). Consistently, using a live cell imaging system, HT22 cells exposed to H_2_O_2_ and a cocktail of ketones (BHB and ACA, 1 mM each) appeared healthy with preserved confluence, compared to cells treated with 200 μM H_2_O_2_ alone for 24 hrs (**Fig. 3D**). Although all ketone treatments enhanced cell viability against oxidative stress, pretreatment with ACA provided more effective protection than BHB alone. Co-application of H_2_O_2_ with a cocktail of ketones resulted in a reduction in cell death (**Fig. 3E**). In contrast, when 200 μM 5HD was co-applied with ketones, neuronal protection against oxidative stress was lost (**Fig. 3E**).

Consistent with our earlier finding that ketones were able to raise ATP levels [3], samples taken from ketone-treated hippocampal slices used in electrophysiological experiments exhibited a slight increase in ATP levels (**Fig. 3F**). Hippocampal slices exposed to 2 mM H_2_O_2_ showed a decrease in ATP levels to 69 ± 7.4% of baseline after 2 hrs exposure, whilst co-application of ketones and H_2_O_2_ restored ATP levels to 94 ± 6.6% of control. These beneficial effects were reversed by 5-HD; ATP levels measured 75 ± 7.1% of baseline (**Fig. 3F**). Overall, these findings strongly support the notion that the metabolic regulation and synaptic protection of ketones is tightly linked through their action on K_ATP_ channels.

### Both mitoK_ATP_ channels and sK_ATP_ channels are necessary for ketone-induced LTP protection

**Fig 4:**
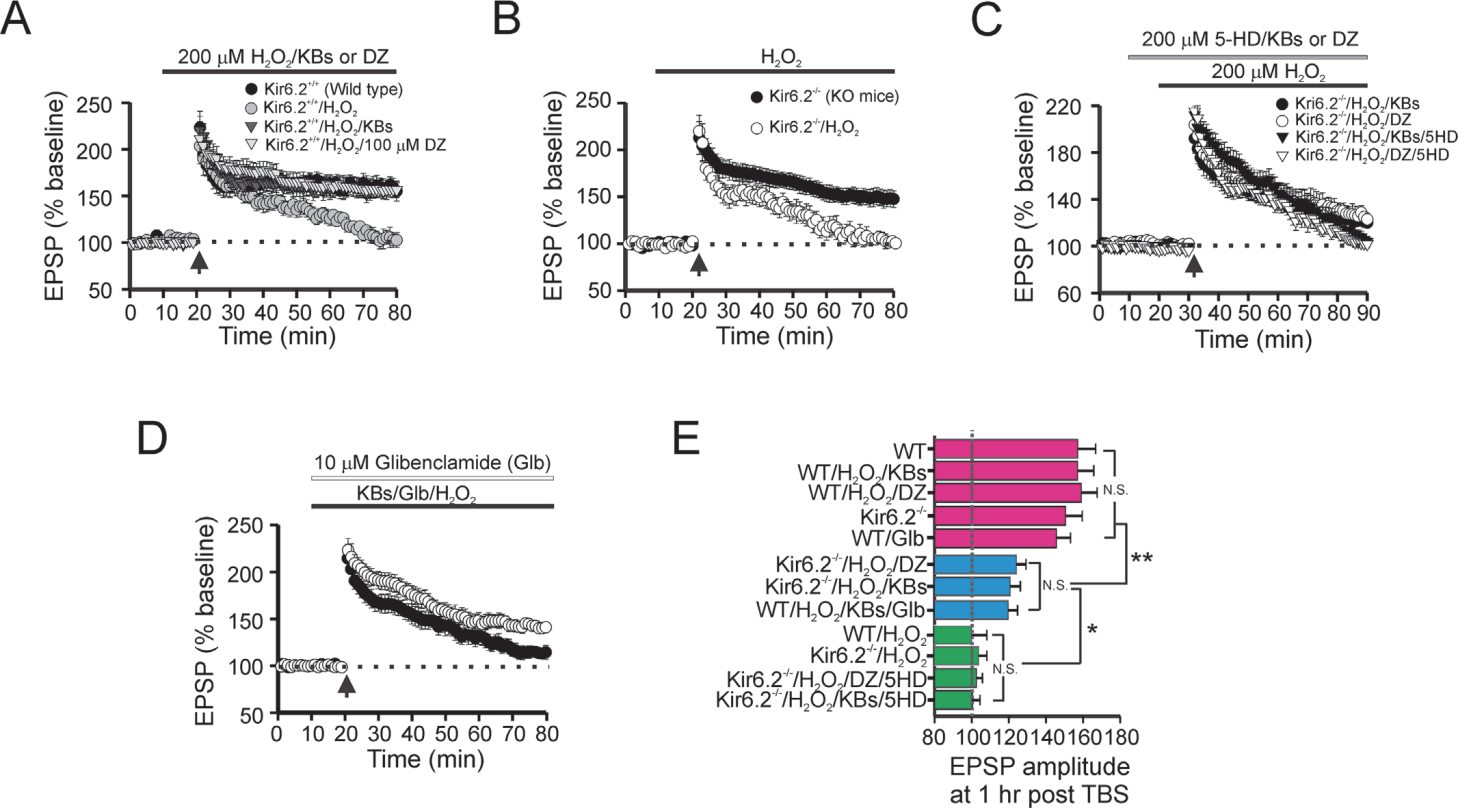
Both mitochondrial- and surface- K_ATP_ channels are necessary for ketone-mediated synaptic protection. (A) TBS-evoked intact LTP in wild–type slices decayed to baseline levels 60 min after H_2_O_2_ application but was maintained in slices incubated with either ketones or DZ (n = 10 slices; 5 WT mice; *** *p* < 0.001). (B) Loss of functional Kir6.2 channels did not affect TBS-induced LTP. However, LTP in Kir6.2 KO slices was impaired by H_2_O_2_ to a basal level indistinguishable from WT slices exposed to H_2_O_2_. (C) Partial blockade of LTP in Kir6.2 KO mice. Changes in LTP from Kir6.2 KO slices were observed in the presence of either ketones (ACA and BHB, each 1 mM) or DZ (100 μM) with H_2_O_2_ (200 μM). In contrast, addition of 5-HD in this condition resulted in the complete blockade of LTP formation (n = 8 slices from 4 KO mice). (D) Pharmacological blockade of sK_ATP_ channels with glibenclamide (Glb; 10 μM) did not significantly alter LTP formation, although it did produce a slight reduction in the EPSP amplitude post-TBS. When Glb was bath-applied, LTP impairment induced by H_2_O_2_ was not fully reversed by ketone application. (E) Bar graph summarizing mean EPSP amplitudes (±SEM) at 60 min after TBS in various treatment groups involving KO mice and WT mice. ANOVA followed by Tukey test; * *p* < 0.05; ** *p* <0.01. N.S. = not significant.

Despite clear evidence that K_ATP_ channels are involved in ketone-mediated synaptic protection, our initial pharmacological experiments with DZ and 5HD did not delineate the relative contributions of sK_ATP_ *vs*. mitoK_ATP_ channels. Hence, to determine which, or if both, channels are involved in the protective effects of ketones, we utilized Kir6.2 knockout (KO) mice lacking the expression of sK_ATP_ channels, but still retain functional mitoK_ATP_ channels [30]. TBS produced robust LTP in slices from wild-type (WT or Kir6.2^+/+^) mice. Hippocampal slices from WT mice exposed to H_2_O_2_ showed impairment of LTP at 60 min following TBS. As with rat hippocampal slices, the LTP deficit induced by H_2_O_2_ in WT mice was reversed by co-application of either ketones or DZ (**Fig. 4A, E**). In Kir6.2^-/-^ mice, TBS produced intact hippocampal LTP (8 slices from 4 mice; **Fig. 4B, E**), and H_2_O_2_ induced a degree of LTP impairment similar to that seen in WT slices treated with H_2_O_2_ (**Fig. 4B, E**). Application of either ketones or DZ along with H_2_O_2_ did not fully preserve LTP formation in hippocampal slices from Kir6.2^-/-^ mice (8 slices from 4 mice; **Fig. 4C, E**). In agreement with our LTP data, working memory deficits in Kir6.2 KO mice were age-dependent manner; disruption was seen at 12 weeks of age but not at 5 weeks [31].

An earlier study showed that pharmacological blockade of sK_ATP_ channels with glibenclamide (Glb) ameliorated LTP formation after high-frequency stimulation [32]. We found that Glb has a tendency to slightly reduce LTP in hippocampal slices of WT mice, but no differences in EPSP amplitude were observed 60 min after TBS amongst these groups (WT, WT/H_2_O_2_/ketones, and WT/H_2_O_2_/DZ; **Fig. 4D, E**). Cotreatment of ketones and H_2_O_2_ was unable to fully preserve LTP when Glb was bath-applied (**Fig. 4D, E**), suggesting that the synaptic protection afforded by ketones might partially involve sK_ATP_ channels. Additionally, the mitoK_ATP_ channel inhibitor, 5HD, completely abolished the partial LTP protection induced by either ketones or DZ in Kir6.2 KO mice (**Fig. 4C, E**). Collectively, these data indicate that both sK_ATP_ and mitoK_ATP_ channels mediate in part the synaptic protective effects of ketones against oxidative stress.

## Discussion

The major finding of the current study is that ketones—at physiologically relevant concentrations—preserve hippocampal synaptic integrity against oxidative stress, likely in part through activation of both sK_ATP_ and mitoK_ATP_ channels. The protective effects of ketones against an exogenous H_2_O_2_ challenge were mirrored by DZ, an activator of sK_ATP_ and mitoK_ATP_ channels, and genetic ablation of sK_ATP_ channels in Kir6.2^-/-^ mice resulted in only partial recovery of hippocampal LTP by ketones and DZ. While a direct action of ketones on K_ATP_ channels could not be demonstrated, similar to earlier studies [19,20,33], our data indicate a key role for both types of ATP-sensitive potassium channels.

It is well known that oxidative stress is a critical factor for the pathogenesis of NDs [34,35]. Oxidative injury induces synaptic impairment, and ultimately cell death, through mitochondrial dysfunction and decreased ATP production, among other actions [3,7,36]. Further, *in vitro* application of H_2_O_2_ results in dose-dependent but site-specific injury (i.e., stratum pyramidale *vs*. stratum radiatum) [37,38]. Although the reasons behind the differential localization-related effects of oxidative stress remain unclear, a major factor may be the resulting region-specific energy deficits that modulate synaptic plasticity [39]. In this context, we found that hippocampal ATP levels correlated well with both cell viability and synaptic integrity as assessed by an established measure of LTP. Specifically, the protective effects of ketones may be related to their ability to enhance ATP production, as well as their antioxidant properties as demonstrated earlier [3,40]. Both of these effects help stabilize the resting membrane potential through preserved Na^+^-K^+^-ATPase function and scavenging of reactive oxygen species (ROS) [41].

Importantly, we show in the present study that pharmacological blockade of K_ATP_ channels with 5HD results in the elimination of ketone-induced synaptic protection and favorable bioenergetic changes in rat hippocampus. Similarly, we observed that 5HD negated the partial protective effects of both ketones and DZ in Kir6.2 KO mice. Collectively, our data support the notion that ketones, through interactions with both sK_ATP_ and mitoK_ATP_ channels, promote mitochondrial homeostasis by enhancing ATP generation and antioxidant activity, consistent with previous studies showing that KD treatment—and possibly the resulting ketosis—elevate cellular antioxidant capacity via improvement in mitochondrial redox status [42,43].

Earlier studies have indicated that ketones may exert neuronal inhibitory effects through sK_ATP_ channels [19,20], likely by increasing their open probability. However, given our finding that ketones increase ATP levels (both in normal hippocampus as well as that exposed to oxidative stress) and that sK_ATP_ channels are known to be inhibited by high ATP/ADP ratios [44], there is an obvious discrepancy that needs to be reconciled. There is abundant evidence in the literature supporting the view that reductions in intracellular ATP/ADP ratios activate sK_ATP_ channels, leading to stabilization of the membrane potential, reduced synaptic excitability and enhanced mitochondrial redox status, all of which contribute to resistance against neuronal damage [19,45–48]. Further, it has been hypothesized that there may be compartmental differences in cellular ATP levels, such that the space immediately subjacent to sK_ATP_ channels may actually have lower ATP/ADP ratios than elsewhere [19]. While the underlying basis for this discrepancy remains unclear, it is possible that ketones may directly bind to and activate K_ATP_ channels, despite cellular ATP levels remaining in a normal or elevated range [20].

Our findings that ketone application in 10 mM glucose-dissolved physiological saline and culture media containing 5.5 mM glucose conferred neuronal protection and synaptic restoration through K_ATP_ channels was somewhat surprising since earlier studies demonstrated that the normal brain glucose detected range of 0.82 to 2.4 mM [49] and the hyperglycemia actually negated the open probability of K_ATP_ channels [50]. On the other hand, Lund et al [51] recently reported that neurons cultured in BHB resulted in a strong alleviation of glycolytic intervention, likely by suppressing glucose metabolism seen with ketones [52]. While there is no direct evidence to elucidate the functional interplay between ATP level and glycolytic activity within ketone application, we cannot exclude the possibility that the decreased ATP supply originating from the glycolysis pathway may be indirectly responsible for enhancing the opening probability of K_ATP_ channels. Alternatively, there is compelling data that K_ATP_ channels can also be activated by various hormones and neurotransmitters through G-protein coupled interactions, cAMP, and protein kinase A [8,9,53]. In this regard, BHB has been shown as an agonist of the G-protein coupled free fatty acid 3 receptor in rat sympathetic neurons [54].

While K_ATP_ channels have been extensively linked to models of NDs and neuroprotective actions, notably as a mechanism for pre-ischemic conditioning [55,56], the functional role of these channels in synaptic neurotransmission remain less clear. However, with respect to cognition, targeted deletion of Kir6.2 results in age-specific disruption of working memory [31]. In Kir6.2 KO mice aged 5–6 weeks, we found intact LTP responses to TBS, but neither ketones nor DZ could rescue LTP impairment caused by oxidative stress in these mice. Clearly, the precise relationships between sK_ATP_ channels and synaptic plasticity during brain development have yet to be clarified [57].

In conclusion, our findings demonstrate that ketones can protect against oxidative impairment of hippocampal LTP, likely through activation of both sK_ATP_ and mitoK_ATP_ channels. These actions subsequently help restore neuronal, synaptic, mitochondrial, and metabolic function. Further, our data underscore the therapeutic relevance of KTX-0101 (a highly ketogenic medium chain triglyceride formulation) in the treatment of AD [4], and of DZ in the long-term treatment of 3xTgAD mice which has been shown to improve learning and memory function, and to reduce anxiety [33]. K_ATP_ channels represent yet another target mechanism that can potently modulate synaptic function, especially under conditions of mitochondrial dysfunction and oxidative stress, both of which have been implicated in various NDs.
